# Racism against racialized migrants in healthcare in Europe: a scoping review

**DOI:** 10.1186/s12939-023-02014-1

**Published:** 2023-09-29

**Authors:** Mia Pattillo, Sigsten Stieglitz, Konstantinos Angoumis, Nora Gottlieb

**Affiliations:** 1 Weill Cornell Medicine, 1300 York Avenue, New York, NY 10021 USA; 2 Bielefeld School of Public Health, Universitätsstrasse 25, 33615 Bielefeld, Germany

**Keywords:** Access to healthcare, Discrimination, Health inequities, Europe, Migration, Racism, Review

## Abstract

**Background:**

Racism is frequently mentioned as a social determinant of migrants’ health and a barrier to health services. However, in the European context, racism and its impact on racialized migrants’ access to healthcare is remarkably under-researched. This scoping review makes a first step toward filling this void by mapping the existing literature on racial and ethnic discrimination against racialized migrants in healthcare in Europe, identifying evidence gaps, and offering recommendations for future research on this topic.

**Methods:**

Following PRISMA guidelines, four databases were searched for empirical studies published in English between 1992 and 2022. Studies were included if they report findings on manifestations, experiences and/or impacts of racial or ethnic discrimination against racialized migrants in a healthcare setting in a European country. They were summarized by study characteristics (geographical scope, study design, research question and measures) and research findings were synthesized.

**Results:**

Out of 2365 initial hits, 1724 records were included in the title/abstract-screening, 87 records in the full text-screening, and 38 records in the data extraction. For many country and healthcare contexts, evidence on racism in healthcare is lacking. Most studies apply an explorative qualitative research design; comparability and generalizability of research results are low. Our analysis furthermore shows a near-exclusive research focus on racism on the interpersonal level as compared to institutional and structural levels. Our synthesis of study results identifies three interrelated ways in which racism manifests in and impacts migrants’ healthcare: 1) general anti-migration bias, 2) health- and healthcare-related prejudice, and 3) differential medical treatment.

**Conclusions:**

Our review underscores how racism reinforces inequities in healthcare access and quality for racialized migrants. It also highlights the need for more research on racism in Europe across a greater scope of country contexts, healthcare settings and migrant/racialized categories in order to understand specific forms of racism and capture race as a context-contingent social construct. It is critical that future research includes the consideration of individual-level racism as embedded in racism on institutional and structural levels. Methods and insights from other disciplines may help to critically examine concepts in light of underlying historical, sociopolitical and socioeconomic processes and structures, and to improve methods for researching racialization and racism in healthcare.

**Supplementary Information:**

The online version contains supplementary material available at 10.1186/s12939-023-02014-1.

## Background

Most European states commit to ensure equitable access to health services and to thus contribute to health equity and the fulfillment of the right to health [1]. However, migrants continue to face persistent inequalities in access to healthcare across European countries [2]. While these inequalities are partly related to restrictions on different migrant groups’ formal entitlements and access to health services, Lebano et al. note that “even when legal accessibility is available, differences and inequalities still exist in accessing healthcare” [3]. Racism is frequently mentioned in the literature as one notable barrier to healthcare for racialized migrants during transit and after arrival in their destination countries [4]. In contexts like the United States of America (USA), the effects of racism on inequalities in health and healthcare have been extensively examined [5, 6]. Based on the existing evidence, prominent scholars have declared racism to be a public health emergency of global concern [7].

In the European context, however, racism and its impact on racialized migrants’ access to healthcare is remarkably under-researched as compared to other informal barriers to migrants’ access to care, such as communication barriers or lack of support in navigating health systems. While there is indeed an extensive body of literature on racial and ethnic disparities in health and healthcare in Europe, their underlying causes and particularly the contribution of racism are insufficiently discussed [8, 9]. Most studies on the health and healthcare of different migrant categories and on migrants’ experiences of healthcare focus on migration status as a determinant, without addressing the compounding effects of being racialized as an inferior Other, and how racialization and racism establish and intensify health inequalities [3, 10, 11]. Hamed et al. demonstrate that there is indeed a dearth of research on racism in healthcare in European contexts in their scoping review of racism in healthcare, which found 67% of the scoped articles to be from the USA, followed by Canada and the United Kingdom (UK) [12].

This limited knowledge may in part be due to the rejection of “race” as a scientific category in many European societies, compared to other countries such as the USA where “race” is a legal category alongside “ethnicity” [13, 14]. Europe’s dismissal of “race” as a legitimate category follows the construction and use of the term as a basis for eugenics and “racial hygiene” most infamously by—but not restricted to—the political, medical and scientific establishment in Nazi Germany. In response to this history, Europe is often constructed as “antiracist”, and racism as an issue of the past, thus hindering productive conversation around this topic in political and public discourse [14, 15].

One consequence of rejecting “race” as a category is that many European countries do not collect related data. Yet, without data on inequalities associated with race/racialization, racism in Europe remains largely invisible [16]. De Genova et al., for instance, advise that “[b]anishing race as a critical analytical category… risks forsaking any adequate account of the distinctly European colonial legacies that literally produced race as a sociopolitical category of distinction and discrimination in the first place” [17]. Hence, while public discussion around racism is common in North America and the UK, European countries have failed to recognize and address racism, reinforcing a tradition of avoiding the topic altogether [18]. As a result, there is a paucity of research on racism in various social arenas, including healthcare, in Europe.

This paper provides an overview of the current empirical research on racism against racialized migrants in healthcare in Europe, and of the role of racism as a barrier to healthcare for racialized migrants. While acknowledging that members of other social categories may also face racism, and that discrimination in other social arenas may also influence healthcare access, we have limited the primary focus of this study to racism in healthcare settings against racialized migrants for reasons of feasibility. To map the respective body of knowledge, we conducted a scoping review of the empirical literature on the topic since 1992. The following sections entail background information on the concept of racism and the state of the art regarding impacts of racism on healthcare, followed by an explanation of our study terminology and methodology. The results section provides an overview of the characteristics of the included literature and a brief synthesis of their conclusions. The paper closes with a discussion of the results of our review, including current evidence gaps and recommendations for future research.

### Racism and racism in healthcare

Racism can be defined as an organized system embedded within a network of social, economic, and political entities in which the dominant racial group, based on an ideology of its own supremacy, categorizes and hierarchically orders groups of people as inferior [19]. Through historical processes of racialization, European regimes divided people(s) into races to strategically create a hierarchy conceived as natural entities in order to justify colonial oppression; i.e., the distinction between different races involved value-judgments about differential capabilities and worth that are unrelated to any biological basis or inherent features [20, 21]. Today, race continues to be a social category—based on nationality, ethnicity, phenotypic or other markers of social difference—which serves to devalue, disempower, and differentially allocate critical resources and opportunities, notwithstanding healthcare [19].

Racism shapes people’s health and healthcare in three interdependent dimensions: the structural (reflecting disadvantaged access to political, economic, physical, and social resources), the institutional (reflecting embedded norms, policies, and practices within institutions that foster discrimination in processes and outcomes), and the interpersonal (reflecting everyday expression/experience of forms of violence that emphasize the devalued status) [22, 23]. The implicit and explicit biases of policymakers, healthcare providers and professionals in other health-relevant positions can affect members of racialized groups on multiple levels, including patient–clinician communication, clinical decision-making, institutionalized practices and policies [24]. It has been evidenced that racial minorities receive fewer medical procedures and poorer quality healthcare than the majority [25]. Various studies have shown that experiences of racial mistreatment in healthcare increase the risk of poor health outcomes, with one of the driving factors being that the expectation of being discriminated against discourages members of racialized groups from accessing health services in a timely manner [26, 27]. People confronted with racism in healthcare thus not only experience the health impacts of disproportionate stress and devaluation, but they also develop mistrust in the healthcare system, preventing the effective use of health services [28, 29]. These issues have been highlighted by the effect of the COVID-19 pandemic on people of color—for instance, multiple studies have demonstrated that structural racism is a key driver of higher SARS-CoV-2 infection and mortality rates amongst Latinx and Black communities in the USA; and anti-Asian xenophobia has contributed to disproportionately high mortality rates among Asian-Americans and Pacific Islanders [30–32].

Despite this paper’s focus on the effects of racism against migrants, it is important and yet challenging to avoid “migrationism”; i.e., the overemphasis of the role of having migrated and the conflation of anti-migrant attitudes and actions with racism [33]. Indeed, while all migrants may experience anti-migrant attitudes, racism will typically target only some (specifically, racialized) migrants. Beyond racialized migrants, members of other social categories in European societies frequently face racism; for instance, members of ethnic minorities such as Sinti and Roma, as well as citizens who may be racialized as an “Other”. To further complicate matters, racism often intersects not only with anti-migrantism but also with other forms of discrimination such as Islamophobia, sexism and classism. Given the aim of this paper to scope the currently existing empirical evidence on racism against migrants in healthcare in Europe, we have limited our focus on research that specifically reports racial or ethnic discrimination against racialized migrants. We do acknowledge that our paper thus fails to capture instances of racism that are not labeled as such by the respective researchers; and that it may moreover fail to do justice to intersecting forms of biases and discrimination in the real-life experience of racism by racialized migrants.

For the sake of conceptual clarity, the following section will provide definitions of the concepts used in this paper and explain terminological choices, before we come to descriptions of our methodology, results and discussion.

### Terminology and definitions

The definition of a migrant varies widely across countries and contexts. As the United Nations Department of Economic and Social Affairs points out, “While there is no formal legal definition of an international migrant, most experts agree that an international migrant is someone who changes his or her country of usual residence, irrespective of the reason for migration or legal status” [34]. For the purpose of this paper, we use this broad definition—including migrants who permanently or temporarily settle in another country, asylum-seekers who are fleeing persecution and violence, and refugees who have been granted asylum. Specifically, our review focuses on racialized minoritized migrants (herein referred to as “racialized migrants”), recognized to be migrants who are racialized as inferior as per processes previously described and who are thus subject to disempowerment, devaluement, and differential access to resources in comparison to the majority population.

Our study focuses on Europe due to its scarcity of evidence regarding racism in various social arenas, including healthcare. For the purpose of our study, we delineate Europe by the fifty recognized sovereign states with territory within geographical Europe and/or membership in internationally recognized European organizations, including but not limited to all countries in the European Union, the European Economic Area, and the UK.

Given that the understanding of what is considered “healthcare” also varies widely across country contexts, we determined healthcare for the purposes of this review to include all physical, dental and mental health services, such as in-patient hospitalization, outpatient clinics, subspecialties, preventative healthcare and screening, and various levels of healthcare administrative and auxiliary services. Consequently, “healthcare users” herein refers to anyone who utilizes the healthcare system as previously defined (including informal caregivers of healthcare users), while “healthcare providers” refers to any staff members who play a role in enabling or hindering the utilization of health services within the previously defined system, including physicians, nurses, midwives, receptionists, medical interpreters, and security personnel.

In the results section of this paper, we will use the terminologies that are used in the reviewed articles to discuss different ethnic and/or racialized groups. Terms such as Black and White will be capitalized to indicate that they are social constructs produced by historical racialization processes.

## Materials and methods

### Study design

We conducted a scoping review to map the geographic scope, study characteristics and focus (research setting, population, question, measures and findings) of the currently available literature on racism against racialized migrants in healthcare in Europe and thus inform further, more in-depth empirical or literature-based research [35]. We followed the PRISMA (Preferred Reporting Items for Systematic reviews and Meta-Analysis) extension for scoping reviews (PRISMA-ScR) [36]; the essential steps of this protocol are described in the following sections.

### Search strategy

We conducted a literature search in four databases (Pubmed, Cinahl, SCOPUS, and Web of Science) in July 2022. It was limited to studies published between January 1992 and June 2022 in English language, and filtered for peer-reviewed articles. The search was limited to the past thirty years for reasons of feasibility; the implications of this limitation are problematized in the limitations section. The search strings were developed in consultation with an academic librarian. They included search terms for the concepts “migrant”, “racism”, “healthcare”, and “Europe”. Search terms and their combinations were adapted to the four different databases (see the search strings for each of the databases in Supplementary file 1). Covidence software (Veritas Health Innovation, Australia) was used to store, organize, screen, and extract the data.

### Inclusion and exclusion criteria

We included a) empirical studies of any design (qualitative, quantitative, mixed-methods), that b) report on racial or ethnic discrimination in healthcare in their results sections, c) focus on racialized migrants as at least one study population, and d) refer to Europe or one of the states in the region of Europe. Exclusion criteria were non-empirical publications (e.g., conceptual articles, commentaries, editorials) and non-peer-reviewed publications (e.g., gray literature, journalistic work), a non-migrant or unclear study population, a different study focus (e.g., on healthcare access generally, without reference to racial or ethnic discrimination as a barrier), a geographical focus other than Europe, a language other than English, and no full-text access. We excluded gray literature, because it comes in a variety of forms which typically have no abstract, posing challenges for data management and extraction as relevance and inclusion criteria cannot be determined without reading the entire piece [37, 38]. Systematic and non-systematic reviews were excluded.

### Screening and extraction

After the removal of duplicates, the screening was carried out in two steps: a) independent title/abstract-screening of all hits by two reviewers each; and b) independent screening of all remaining full-texts by two reviewers each. In both steps, the first author independently screened all titles and abstracts to assess eligibility for inclusion, and two other authors (KA, SS) split the total number of articles to screen. Conflicts were resolved by discussion among all authors.

The included articles were summarized by year of publication, country context, research goal, methodology, and main findings. An overview of the summaries of all 38 articles can be found in the supplementary material (Supplementary file 2).

An extraction sheet was developed, based on the consolidated criteria for reporting qualitative research (COREQ) and the STROBE items for reporting observational studies in epidemiology [39, 40]. In accordance with the aims of this scoping review, the extraction focused on study designs and characteristics, geographical focus, study populations and questions, measures of racism, and key takeaways regarding racialized migrants’ exposure to racism, and the role of racism for racialized migrants’ access to healthcare. After piloting, the final extraction sheet was applied to all included articles by MP, with review by KA and SS.

### Quality assessment

The quality of the included papers was assessed by two raters (KA and SS) using the critical appraisal tools of Joanna Briggs Institute (JBI). A score of 80% was set as a benchmark to determine a high level of evidence; a score of under 40% indicated a low level of evidence. The included qualitative studies were assessed with JBI’s checklist for qualitative studies [41]. We dichotomized each of the ten questions to yes (1 point) or no (0 points), giving a scale ranging from 0 (poor quality) to 10 (high quality). The included quantitative studies were assessed with JBI’s checklist for analytical cross-sectional studies which includes seven scales [41]. Questions were dichotomized in the same way as the qualitative questions, with a scale ranging from 0 (poor quality) to 7 (high quality). The n = 1 included mixed-method study was evaluated by using both checklists for the different parts of the study, and is charted under the qualitative studies as this was the article’s methodological main focus.

A pilot quality assessment was done to increase inter-rater reliability. After the pilot screening, KA and SS divided the qualitative and quantitative studies, and each assessed half. In case of uncertainties both raters discussed and decided together. The results of the quality assessment in terms of the included studies’ level of evidence is included in Table 1 below.

**Table 1:** Overview of study designs, sampling methods, level of evidence, and healthcare settings

	Qualitative *n* = 27	Quantitative *n* = 10	Mixed-Methods *n* = 1
*Study design & method*			
Interviews	23 (85%)		1 (100%)
Focus groups	8 (30%)		
Ethnographic observations	2 (7%)		
Retrospective		5 (50%)	
Cross-sectional		4 (40%)	1 (100%)
Prospective		1 (10%)	
*Sampling*			
Population-based		4 (40%)	
Non-population-based	27 (100%)	6 (60%)	1 (100%)
*Level of evidence*			
High	19 (70%)	4 (40%)	1 (100%)
Medium	8 (30%)	4 (40%)	
Low		2(20%)	
*Settings*			
Healthcare broadly, or primary healthcare	10 (37%)	6 (60%)	
Reproductive healthcare	7 (26%)		1 (100%)
Mental healthcare	4 (15%)		
Dementia	1 (4%)	1 (10%)	
Gastroenterology		2 (20%)	
HIV/AIDS	2 (7%)		
Chronic illness	1 (4%)		
Geriatric	1 (4%)		
Palliative care	1 (4%)		

## Results

**Fig. 1:**
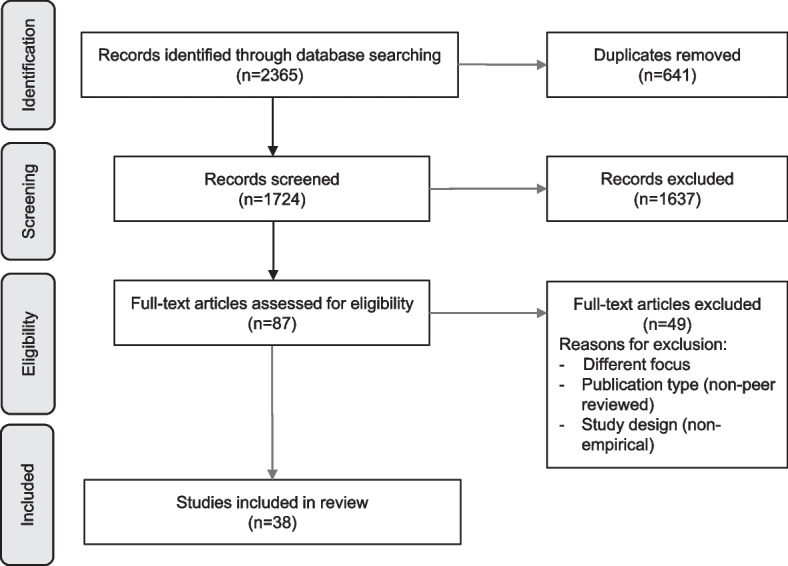
PRISMA flow-chart

The initial search in the four databases yielded a total number of 2365 articles. After de-duplication, 1724 studies were included in the title/abstract screening. Out of these, 87 texts were read in full for eligibility. In the full-text screening, 49 full-text articles were excluded due to either (a) a different research focus, (b) the publication type, or (c) the study design. Ultimately, 38 articles were included in the data extraction. The screening process is presented in Fig. 1.

The following section begins with a broad overview of the descriptive characteristics of the 38 included studies, followed by a synthesis of their study questions, measures and main results.

## Characteristics of the included studies

### Geographical scope

Eight (21%) of the 38 included studies stem from the UK, including overall UK (3), England (3) and Scotland (2). The remaining 29 studies were conducted in the following country contexts: Norway (5), Belgium (4), Sweden (4), France (3), Germany (3), Finland (2), Netherlands (2), Switzerland (2), Denmark (1), Ireland (1), Portugal (1), and Spain (1). One study looked across 10 EU countries.

### Study designs

Of the 38 articles in this scoping review, 27 (71%) articles were qualitative studies, 10 (26%) articles were quantitative studies, and one article employed mixed-methods. The 27 qualitative studies included focus groups, interviews, and/or ethnographical observations. Of these, 18 articles involved only healthcare users (or informal caregivers of healthcare users) as participants, four articles involved only healthcare providers as participants, and five involved both healthcare users and healthcare providers.

The 10 quantitative studies included four cross-sectional surveys, all of which surveyed healthcare users. Five articles were quantitative studies evaluating differential treatment between native and migrant populations based on patient data. One article measured implicit and/or explicit bias among healthcare providers. The sample size for these quantitative studies ranged from 57 to 3,382,394, with a median sample size of 1,127. One article was a mixed-method study, which comprised qualitative interviews with 33 Sub-Saharan African women and analysis of data from their medical files.

### Sampling methods

The qualitative studies recruited participants via purposive sampling [42–46], quota sampling [47], and convenience sampling, which was typically followed by snowballing. Convenience sampling involved the acquisition of participants from various clusters, including healthcare and nursing institutions [48–55], immigrant associations, non-governmental organizations, religious institutions, or community centers [54, 56–63], specific illness support groups or workshops [64, 65], specific cities or geographical areas [66–68], and/or universities [69]. The mixed-method study employed convenience sampling within cluster sampling by inviting women amongst three public maternity units to partake in the study [70]. Hence, a large part of the included qualitative studies relates to specific contexts such as particular healthcare facilities or non-governmental organizations and a majority of studies are prone to selection bias and/or volunteer bias from convenience and snowball sampling.

Of the quantitative studies, all reviews of medical files employed probability sampling methods, with files taken from specific hospitals, specific departments within hospitals, or region or country-wide health registers [71–76]. Studies that employed cross-sectional surveys of healthcare users included data from the Health 2011 survey data representative of the country of Finland [77], cluster sampling by administering a questionnaire in 16 language schools across four counties of Sweden [78], simple random sampling via a large-scale, nationally representative country-wide survey [79], and a voluntary questionnaire sent out to migrants across ten European countries recruited from points of healthcare delivery services followed by selection via snowball technique [80].

### Research questions and measures

Most of the studies report on racism in general healthcare [42, 43, 49, 50, 54, 60–63, 69, 77–82] or in relatively broad contexts such as primary healthcare [42, 43, 49, 50, 54, 59–63, 69, 77–82]. Some articles examine experiences of racism within specific healthcare settings or experiences related to specific treatments or illnesses. Among these works, reproductive health was the most frequent focus. Out of eight studies, two studies looked at healthcare for childbearing women [45, 57] and one study each looked at care delivered by midwives [48], care during pregnancy and delivery [58], maternity services [53], cervical screening [44], prenatal care for women with hypertensive disorders [70], and reproductive health [68]. Mental health was another frequent focus. Out of four studies, one study each looked at mental health and addiction services [43], mental health services [47], outpatient treatment for depression [74], and the use of restraint in emergency psychiatry [75]. Two studies focused on dementia, with one study looking at care for healthcare users with dementia [67], and another looking at quality of diagnostic evaluation of dementia [76]. Two studies lay in the field of gastroenterology, including one study looking at care for healthcare users with ulcerative colitis [72], and another looking at care for patients with inflammatory bowel disease [73]. Another two studies focused on the care for persons living with HIV/AIDS [64]. Further studies examined racism in care for chronically ill adults [66], care for elders [52], and palliative care [46].

Some studies alluded to migrants as a study population generally or studied migrants across a range of countries and regions; whereas other studies focused on migrants from specific regions or religious backgrounds. The most common region of origin for racialized migrants in the included studies was Africa, with a total of 10 articles, including two articles that studied healthcare for migrants from African countries broadly [63, 65], six articles on migrants from Sub-Saharan Africa, [51, 54, 60, 61, 64, 70], one article including migrants from Ethiopia [62], and one article including migrants from Morocco [67]. Three studies focused on migrants from European and Middle Eastern countries, including one on migrants from Eastern European countries broadly [73], one on migrants from Syria [42], one on migrants from Turkey [58, 66], and one on migrants from Turkey and Portugal [58]. The study looking at migrants from Eastern Europe also looked at migrants of Afro-Caribbean descent [73]. There were three studies that focused on migrants from Asia, including two studies on migrants from South Asia [72] and one on migrants from China [47].

### Comparisons across country contexts and ethnic/religious backgrounds

Only two of the included studies compare findings across geographical regions and ethnic/religious backgrounds to build an understanding of which categories of racialized migrants experience greater degrees of perceived discrimination, and where. One cross-sectional study assessed discrimination within healthcare settings as perceived by racialized migrants in 10 European countries (Austria, Bulgaria, Cyprus, France, Germany, Greece, Italy, Malta, Spain, and Sweden). A high level of perceived discrimination was reported by those who had migrated to Greece, Italy, Cyprus, and Austria, in comparison to the other included countries. The study furthermore purports that migrants from Afghanistan and Iran tend to perceive/report racial discrimination more than participants from other included countries such as Iraq, Nigeria, and Syria [80]. The second survey examined social disparities in discrimination within healthcare and foregone healthcare amongst migrants in France. It suggests that rates of both reporting discrimination within healthcare and reporting foregone care were generally highest amongst migrants from African countries and Overseas France (as compared to migrants from European and Asian countries). (Overseas France, a term used in the study, refers to 13 French-administered territories outside of Europe, which have remained of the French colonial empire; e.g., Martinique and French Guiana) [79].

Our review pinpoints high variability in the design, methods, and measurements of the included studies. As shown in Table 1, four out of 10 quantitative studies scored 80% or higher in the quality assessment. Most studies were lacking strategies for coping with identified confounders. Among the qualitative studies, 19 out of 27 studies scored 80% or higher in the quality assessment. What was lacking in 23 out of 27 studies was locating the researcher culturally and theoretically. Only 13 studies addressed the researcher's influence on the research and vice versa. Apart from that, all studies adequately represented the participants as well as their voices, and showed a clear and cohesive relationship between analysis and conclusions.

### Manifestations and impacts of racism on healthcare

The following sections summarize the findings of the included studies with regard to the manifestations of racism against racialized migrants in healthcare settings and the effects of racism on healthcare for racialized migrants. Our analysis identified three main themes, which describe different—albeit interrelated—ways in which racism shows in healthcare settings. The first theme refers to healthcare providers’ expressions of general (i.e., not specifically health- or healthcare-related) anti-migration bias, Othering, and racist language and behavior during the process of health service provision. The second theme comprises specifically health and healthcare-related prejudice; and the third theme relates to the provision of differential medical care for racialized migrant healthcare users. The three themes frequently interrelate in unfolding their impacts on healthcare access and delivery for racialized migrants. Evidence pertaining to migrants’ responses to racism in healthcare and related effects on healthcare seeking are summarized in a fourth subsection.

### General anti-migration bias, Othering, and racist language and behavior

Several studies found implicit or explicit anti-migrant attitudes among healthcare providers, including resentment toward the utilization of healthcare services by racialized migrants. For instance, a study on implicit and explicit ethnic bias among general practitioners in Belgium found that the majority of participants had implicit preferences for Belgian healthcare users to the detriment of racialized ethnic and migrant groups [82]. Othering was another frequent form of implicit bias, manifesting in the language that healthcare providers use to speak about migrant healthcare users. This was demonstrated, for example, in a study on maternity care services in Ireland: The language used by healthcare providers evoked “Us versus Them” comparisons as it set Irish women against racialized migrant women, with the latter being homogenized as African/Black [53].

Overt display of racism by healthcare providers through language and behavior was reported in multiple studies, including studies with Black African asylum-seekers in Germany, with South Asian Sikh and Muslim healthcare users in Scotland, and with Black African asylum-seekers in France, all of whom attributed their experiences to their ethnic background and related markers [46, 55, 63]. For instance, a Turkish woman in a Swiss study reported being called a “Mora,” a derogatory term for Muslims [57, 58]. In a Belgian study, the informal caretaker of a racialized migrant healthcare user described a healthcare provider talking to him and his father “like we were animals…it hurts me that my father has to endure such racism at this stage of his life” [67].

In several studies, racialized migrant participants described being asked intrusive questions, which perpetuated their devalued identity and led to the feeling that they were unfairly scrutinized [61, 63]. For instance, a participant in a Norwegian study recalled being interrogated by healthcare providers about “what brought me to this country and why don’t I go back to Uganda and find a better job” [61]. A study from Scotland reports that, when an asylum-seeker with multiple long-term conditions questioned the medical treatment and food he was provided, he was met with hostility by nurses, one of whom said: “I will paint a horrible picture of you and report you to the immigration and they will deport you” [46]. Participants in other studies described similar threats or comments targeting their vulnerable status as migrants, including a patient who was told that he was being taken care of as a favor, and that “[he] had to do [his] part of the job and contribute to social security once [he] had acquired the residence permit” [60].

Instances of overt racism against healthcare users were also testified by healthcare providers. In a study conducted in Ireland, for instance, maternity service providers described interactions that they felt to be “unacceptable and racist” from both other staff and patients in the hospital [53]. In a German study, several physicians caring for chronically ill Turkish adults reported witnessing incidents of racism toward older adults of Turkish descent in the healthcare system [66].

### Health and healthcare-related prejudice

Several interview studies suggested that racialized migrants also frequently face health-related prejudices, often specific to their ethnic or national category. For instance, an Indian parent requesting a prescription for infant formula was challenged, “Why do you want infant formula for your child? In India they don’t drink milk” [42, 55]. The majority of stereotyped interactions were reported by studies with Sub-Saharan African and Black healthcare users. The most common stereotype described was that migrants from African countries were carrying infectious diseases into their destination countries [60, 64]. For example, a study participant living with HIV/AIDS in Belgium pointed out that, even in healthcare settings, HIV was still perceived as a foreign disease and Africans were blamed for its spread [64]. Similarly, in a French study, a participant from an African country recounted being told by a nurse that refugees “brought diseases like polio that had disappeared in Europe a long time ago” [60]. This perception also manifested in the behaviors of healthcare providers, such as facial expressions upon direct skin contact and double gloving [61]. The study participants unambiguously established how such discriminatory labels were rooted in racism and coloniality, which set Black people apart from Whites by stigmatizing them as “animals, uneducated, rude, primary, ignorant,” or inherently “bad and criminal” [54, 63].

Studies with healthcare providers further alluded to the fact that negative perceptions of providing care for racialized migrants are widespread among this group. In an Irish interview study, healthcare providers expressed negative feelings when talking about racialized migrant healthcare users, with multiple interviewees repeating the words “stress”, “worry”, “difficult”, “frustration”, and “tired/exhausted.” Many healthcare providers did not consider themselves racist; they felt their issues were valid: “Because they’re in our face all the time and they’re rude to us, … I’m not racist, it’s very frustrating” [53].

Oftentimes, negative perceptions of providing healthcare for racialized migrants were based on generalized biases against this group, such as the idea that they exaggerate their pain or that extrinsic control and force are warranted to ensure treatment compliance [52, 53]. Physicians participating in a Swedish study commented that racialized migrants over-emphasize their symptoms. They moreover expressed concern that migrant patients did not follow treatment regimens correctly, and that changing their habits “requires a bit more force” [48]. One physician caring for chronically ill Turkish adults in Germany stated: “It’s exhausting… Let’s say a female patient of Turkish descent comes in and she’s quite hysterical – in my perception. Then it would take me ten minutes to first of all talk her down from that state of hysteria. That’s really a lot of work for me. And when I feel stressed, it makes me aggressive” [66]. The statement exemplifies interlocking levels of discrimination, as the concept of the “hysterical migrant patient” has a clearly gendered undertone intersecting with its racist connotation. Such findings further illustrate how the above described biases tend to deny racialized migrant patients’ credibility, rationality and responsibility; and how they compromise the caregiver-patient interaction and healthcare delivery, linking this aspect to the aspect of “differential medical treatment” (see following subsection).

Some healthcare providers acknowledged having witnessed racism on behalf of their colleagues. Participants in a Norwegian study, for instance, reported witnessing other healthcare providers talking behind racialized migrant healthcare users’ backs and stigmatizing women with racialized migrant backgrounds, particularly Sub-Saharan African women [51]. In a Swedish study, a physician described interpersonal discrimination against racialized migrant patients and reasoned that this occurred “due to nursing staff that are not open minded” [56].

### Differential medical treatment

Several studies cited lack of responsiveness during healthcare delivery as a frequent experience among racialized migrant healthcare users. Migrants related this differential treatment to healthcare providers’ negative attitudes toward racialized migrants and people of color. Several studies noted longer waiting times for appointments for racialized migrant healthcare users as compared to native patients [50, 68]. Migrant participants in a Norwegian study felt that doctors dismissed them while being warmer toward Norwegian patients “because some of them don’t like non-Norwegians or Africans” [63]. In another study, a participant noted that the nurse ignored them during dialysis and instead turned their attention to non-migrant patients; and another participant said that, “They pretend not to understand you and they ignore your presence and concentrate on different patients that are White” [61]. As one Black African asylum-seeker in a German study said, “Some of the doctors, especially when you are Black and a foreigner, they don’t care” [62]. In several studies from various country contexts, racialized migrant women describe similar experiences during pregnancy and maternal care. For instance, Syrian women in Germany felt that their health complaints were not taken seriously and that they were not properly examined [42, 43, 61]. Asylum-seekers in two separate studies conducted in France and Germany both described refusal of care to which they were entitled [60, 63].

Multiple studies reported migrant healthcare users’ experience of differential medical treatment and lower quality of care [47, 54]. They include a Swedish study, in which a participant with epilepsy explains that they were never offered a dietitian when a non-migrant friend was offered one right away. Most participants in the same study wondered if the treatment approaches offered to them were affected by their migrant status [56]. In another study in the UK, participants living with HIV expressed the sentiment that Black African patients were not well looked after, manifesting, for instance, in the neglect of dietary needs [65].

In several studies, healthcare providers also described witnessing differential treatment. A health professional working with patients with life-limiting illness in Scotland confirmed that a racialized migrant healthcare user had received poor care and that his dietary needs had not been met, on top of generally discriminatory treatment, with other clinicians suggesting “that they send him [the healthcare user] back [to his country] as soon as they possibly could, apparently without any notion of the consequences” [46]. Multiple midwives interviewed for a Swedish study noted that racialized migrant women did not receive the same treatment and care as native Swedish women, perhaps due language barriers, or healthcare providers’ “ignorance or prejudices” [48].

Quantitative studies based on patient files confirm differential treatment between racialized migrant and non-migrant healthcare users. One retrospective analysis of the treatment of ulcerative colitis in England found that patients of South Asian origin received poorer quality clinical care, were significantly less likely to be reviewed by a consultant, and were more likely to be discharged than their European counterparts. Moreover, South Asian patients were admitted to hospital more often but had significantly fewer tests than European patients [72]. Another study conducted on patients with inflammatory bowel disease in England found that Afro-Caribbean healthcare users were at significantly higher risk of not receiving treatment compared to White British healthcare users; and South Asian healthcare users were less likely to be admitted to hospital [73]. A study conducted on outpatient treatment for depression in the Netherlands found that timeliness and treatment intensity were less favorable for Moroccan, Turkish, and other non-Western healthcare users compared with ethnic Dutch [74]. A study conducted in a department of acute psychiatry in Norway found that patients with immigrant background were both more often and more heavily restrained, while another found significant differences in the quality of the diagnostic evaluation between migrant and non-migrant patients [75] A study conducted in France suggested non-medically justified differential prenatal care between women from African countries and non-migrant women, including blood pressure measurement procedures, screening tests and failure to act upon borderline values [70].

### Migrants’ responses to racism and impacts on healthcare access

As the above described research findings indicate, racialized migrant healthcare users are aware of the racist bias and discrimination that they may face in healthcare settings. In response, they express pain and distrust, and tendencies to avoid or delay healthcare seeking. In a Swedish study among asylum seekers 10.9% of the respondents indicated experiences of feeling offended or insulted by racialized language or remarks from medical staff and expressed distrust in healthcare providers [78]. Racialized migrant women in a Swedish study expressed that they felt uncared for or ignored during pregnancy, miscarriage, and childbirth; and they stated that they were treated as if they were exaggerating, legally incompetent, or immature [45]. The frequent failure to provide adequate attention to racialized migrant healthcare users led many to express feelings of being neglected and discriminated against, being treated as second-class patients, or not being seen as a person at all [61]. In several studies, racialized migrants mention being collectively viewed as overly demanding, complaining, and “something strange” by healthcare providers as an important barrier to seeking health services [46, 49, 57]. These results underscore how racism reinforces inequities in healthcare access, which may eventually contribute to differential health outcomes for racialized migrants.

## Discussion

This scoping review aimed to map the current empirical evidence on racism against racialized migrants in healthcare in Europe. One major insight from our review pertains to the scarcity of generalizable evidence on racism in healthcare in Europe, which is related to the geographical scope, study designs and methods of the existing research. The 38 included studies stem from 14 different country contexts across Europe. For most European countries, little to no evidence is available (even though this can partially be attributed to the fact that our search only included articles in English, see limitations section below). To gain a more comprehensive picture of the extent and impacts of racism against racialized migrants in healthcare in Europe, the collection of more data on racialization and racism will be an indispensable first step. In some contexts, this will require the acknowledgement of racialization and racism as social realities and key determinants of health inequities—intersecting with other determinants such as migration status, gender and socioeconomic position—and the consequent recognition of racialization as a valid and important analytical category [83].

Only two of the included studies compare across geographical regions and migrant categories. These comparative studies offer insights into whether and how individuals and groups are differently constructed as “Others” and discriminated against as such in different contexts. Yet, differences in study designs (e.g., a focus on different migrant categories) make it difficult to draw conclusive insights. Therefore, there is a particular need for systematic comparative studies, given that they have the potential to make salient contributions to the study of racism/racialization as a socially constructed and contextualized phenomenon [84].

Moreover, qualitative studies make up the majority of the identified literature. Qualitative research is important for exploratory research and hypothesis generation. It provides critical insights into the experiences of racism in healthcare by healthcare users and providers, and the meaning of these experiences for them. However, qualitative studies are limited in that they cannot generate generalizable insights into quantifiable aspects of the phenomenon. For instance, how frequent is racial discrimination in healthcare settings? Where and when does it occur? Who is targeted? Such measuring requires quantitative research with representative samples. Yet, half of the quantitative studies included in this review employ non-probabilistic sampling methods; many acquire their data from specific settings such as particular healthcare settings – namely inpatient healthcare, reproductive or mental health – or particular healthcare facilities. This may render the respective studies prone to selection and social desirability bias (for instance, if those healthcare facilities that allow research on racism within their premises tend to display greater willingness to acknowledge and address related issues; or if healthcare users in certain healthcare facilities tend to avoid offering negative evaluations of the services received, for fear of consequences for their healthcare). It also implies that many healthcare settings as well as various levels of healthcare administrative and auxiliary services (for example, receptionists and security services) have barely been examined at all.

Another central insight from our scoping interview is that most research in this area focuses on racism on the interpersonal level, while evidence of racism on institutional and structural levels remains extremely scarce. The current scope of research may thus run the risk of obscuring wider sociopolitical racialization processes and the ways in which racism is systematically reproduced and normalized in healthcare settings, inter alia through health policies, the design and organization of healthcare systems, health information and communication, biomedical norms, technologies, and—importantly—research itself [85, 86]. This finding resonates with existing scholarship, which claims that the effects of structural racism are often presented through measurements of interpersonal discrimination. The resulting overemphasis of the role of individual perpetrators contributes to a simplistic framing of racism in healthcare as a problem of “a few bad apples” [87]. Such framing, in turn, tends to relieve institutions, authorities and society of any responsibility by purporting individual-level interventions instead of systemic changes [88]. Hence, it is important to generate evidence on and to counteract racism on interpersonal levels, but this must be complemented by research and interventions that address racism as a structural phenomenon [89, 90].

Yet, our review points to current conceptual and methodological difficulties in the operationalization of racism on institutional and structural levels (as opposed to racism on interpersonal level), and the coping with intersecting biases such as anti-migrantism, Islamophobia, sexism, and classism—both in the included studies and in our own research. In regards to the operationalization of institutional and structural racism, for example, our search strategy implied the exclusion of publications that report differential health policies or inequalities in access to healthcare without specifically naming racism as a factor. This decision may have led to the omission of relevant research (see limitations section below). The alternatives would have been either to include all publications on healthcare inequalities for migrants, thus interpreting all differential health system outcomes for racialized migrants as a function of racism; or to make case-by-case judgments whether the reported research findings are related to racism or not, which would require clear and actionable criteria to do so.

In regards to coping with intersecting bias and disadvantage, many studies included in this review did not aim to study racism; rather, issues of racism were brought to light within conversations on the broader challenges faced by migrants in obtaining health services. The scarcity of research that focuses specifically on racism in healthcare indicates low attention for this issue among the academic community; it may also imply that racism in healthcare is more prevalent in Europe than the current evidence suggests, simply because “[i]f you don’t ask, you don’t know” [91]. From the currently available body of evidence, it is thus often challenging to parse out findings related to racism from those that are related to other, potentially intersecting bias and disadvantage (as also shown by the results of our quality assessment, which pointed to frequent deficiencies in coping with confounders). This continues to reproduce failures in identifying and examining racism, conflating it with other factors at play. Such problematics in currently existing research underscores the need for conceptual discussion and the development of actionable concepts and tools for research on racism.

Given a growing awareness of the importance of collecting data on migration, racialization and racism, for example, in the German context [92–94], better concepts and methods may bolster further research to systematically fill the aforementioned evidence gaps regarding racism in healthcare in Europe. Comparative analyses in and across additional country contexts, among different migrant (and non-migrant) categories, and in different healthcare settings will be important—not to create a hierarchy of victimization, but to understand the specific forms of discrimination that may be associated with the respective stereotypes and settings. Moreover, they will capture racialization as a context-contingent social construct [95]. In doing so, future research on racism in healthcare can vitally benefit from interdisciplinary and participatory approaches. Methods and insights from other disciplines and social arenas such as education, labor market integration or housing, will facilitate conceptual and methodological development; and a genuine diversity of perspectives will help to critically examine categories and hierarchies—including “both sides of the coin”, disadvantage and privilege—in light of underlying historical, sociopolitical and socioeconomic processes and structures [12, 96].

Part of this discussion will need to focus on the risks of research in reproducing the very racialization processes that it wants to examine, despite best intentions [97]. Our quality assessment indicates frequent shortcomings in researchers’ positionality. A large part of the studies included in this review focus on migrants from African and Middle Eastern countries and on migrants of Muslim faith. In terms of healthcare settings and needs, many studies focused on (female migrants as) pregnant women and mothers, and on healthcare users with mental illnesses. While there are various justifications and explanations for such patterns, we would also like to emphasize the need for continuous critical reflection on the role of research, its embeddedness in institutions and structures of racial inequality, and the extent to which it may thus contribute to stereotyped representations of some migrant categories (for example, as passive and vulnerable) and to their problematization.

All of the above challenges notwithstanding, our review did identify common themes concerning the manifestations and impacts of racism against racialized migrants in healthcare settings. Racism manifests through Othering, stereotyping, lack of responsiveness and dismissal of symptoms; healthcare providers also express overt racism through offensive language and behaviors. Moreover, the quality of the medical care provided for racialized migrants often differs from that of non-migrant healthcare users. Racialized migrants themselves recognize racism and differential treatment. In response, they express distrust in healthcare providers and the healthcare system, and they may tend to delay or forego medical treatment [78, 98]. These results underscore that racism reinforces healthcare-related inequities, which may eventually contribute to inequities in health outcomes for racialized migrants.

### Limitations

It cannot be ruled out that our scoping review failed to identify potentially relevant literature; for example, if it was not published in English language or if it was not published between 1992 and 2022. Our search strategy may thus have skewed our analysis toward contexts that use English as official or academic language, while failing to capture the results of older studies on racism which may have revealed trends in the study of racism over the years. Nevertheless, we feel that our review is valuable in beginning a conversation on the topic of racism in healthcare in Europe, and in describing the characteristics and content of the evidence that is accessible to a broad audience, particularly given the fact that English is a widely spoken and read language. Furthermore, as explained earlier (see section Racism and racism in healthcare), our review may have failed to capture relevant research if it did not report findings related to racism as such. While other forms of discrimination such as anti-migrantism and Islamophobia often intersect with racism, we excluded papers on these topics unless they also reported findings specifically related to racial or ethnic discrimination. This methodological decision may have led to an erasure of instances of racism against racialized migrants that do not appear as overtly, and it may thus reproduce a rather narrow understanding of racism that disregards the intersectional character of manifestations and experiences of racism in reality. Despite these limitations, we believe that our paper does provide an important first overview of the research landscape on racism against racialized migrants in healthcare in Europe, which can inform future research efforts.

## Conclusions

This review maps the empirical evidence of racism against racialized migrants across a variety of healthcare and country contexts in Europe. It shows how racism reinforces inequities in healthcare access and healthcare quality, inter alia through overt and covert displays of racism by healthcare providers and through compromised quality of diagnosis and treatment. As indicated by previous research, experiences of racism perpetuate distrust among racialized migrants and discourage them from accessing health services when needed [26]. Intersecting with other determinants of health inequities, racism in healthcare may thus contribute to poor health outcomes among racialized migrants and other racialized social categories. Informed interventions to address racism in healthcare are therefore imperative to mitigate health inequities.

However, our review pinpoints major gaps in the current knowledge on how racism manifests in and impacts healthcare in Europe, particularly across geographical contexts and healthcare settings. The overall paucity of data, differences in study designs and a tendency to rely on setting-specific sampling approaches limit the generalizability of the extant evidence. In addition, the current research focuses on interpersonal-level racism while largely failing to consider embedded structural levels and processes; namely racism on institutional and structural levels as well as underlying historical and sociopolitical racialization processes.

A more comprehensive and nuanced understanding of racism in healthcare will require further research to generate systematic and robust data across different country and healthcare contexts and racialized social categories. Such research will benefit from inter- and transdisciplinary collaborations, in that conceptual, methodological and practice exchange will help ground public health research in theory, including critical reflection of the role of health research in reproducing racialized assumptions and norms.

### Supplementary Information

**Additional file 1:** **Supplementary file 1.** Search terms and search strings.

**Additional file 2: Supplementary file 2.** Overview of the included literature.

## Data Availability

Not applicable.

## Abbreviations

UN: United nations
EU: European union
UK: United Kingdom
USA: United States of America
