# Supplementary material for: Racism against racialized migrants in healthcare in Europe: a scoping review
Source: Int J Equity Health. 2023 Sep 29;22:201. doi: 10.1186/s12939-023-02014-1 (PMC10540333; doi:10.1186/s12939-023-02014-1)
Supplement: Supplementary file 1 — Additional file 1: Supplementary file 1. Search terms and search strings. [file 12939_2023_2014_MOESM1_ESM.docx]

**​​Supplementary file 1: Search terms and search strings**

**Search Strings:**

**Migrant:** (refugee* OR "asylum seeker" OR asylum OR migrant* OR "forced migration" OR migration OR immigra* OR stateless OR "state-less" OR "residence status" OR "foreign-born" OR "noncitizen" OR outsider OR newcomer OR "newly arrived" OR "new arrival" OR "recent entrant" OR "non-national" OR "transient" OR "Refugees"[MeSH Terms] OR "Human migration"[MeSH Terms] OR "Transients and Migrants"[MeSH Terms])

**Racism:** (racism OR racial OR racialization OR racialisation OR discrimination)

**Healthcare:** (healthcare OR "dental care" OR patients OR "health care" OR hospitals OR "emergency service" OR health OR "medical staff" OR "health staff")

**Europe:** (Europe OR European OR Germany OR "United Kingdom" OR France OR Italy OR Spain OR Ukraine OR Poland OR Romania OR Netherlands OR Belgium OR "Czech Republic" OR Czechia OR Greece OR Portugal OR Sweden OR Hungary OR Belarus OR Austria OR Serbia OR Switzerland OR Bulgaria OR Denmark OR Finland OR Slovakia OR Norway OR Ireland OR Croatia OR Moldova OR Bosnia OR Herzegovina OR Albania OR Lithuania OR "North Macedonia" OR Slovenia OR Latvia OR Estonia OR Montenegro OR Luxembourg OR Malta OR Iceland OR Andorra OR Monaco OR Liechtenstein OR "San Marino" OR "Holy See")

**PubMed:**

(refugee* OR "asylum seeker" OR asylum OR migrant* OR "forced migration" OR migration OR immigra* OR stateless OR "state-less" OR "residence status" OR "foreign-born" OR "noncitizen" OR outsider OR newcomer OR "newly arrived" OR "new arrival" OR "recent entrant" OR "non-national" OR "transient" OR "Refugees"[MeSH Terms] OR "Human migration"[MeSH Terms] OR "Transients and Migrants"[MeSH Terms]) AND (racism OR racial OR racialization OR racialisation OR discrimination) AND (healthcare OR "dental care" OR patients OR "health care" OR hospitals OR "emergency service" OR health OR "medical staff" OR "health staff") AND (Europe OR European OR Germany OR "United Kingdom" OR France OR Italy OR Spain OR Ukraine OR Poland OR Romania OR Netherlands OR Belgium OR "Czech Republic" OR Czechia OR Greece OR Portugal OR Sweden OR Hungary OR Belarus OR Austria OR Serbia OR Switzerland OR Bulgaria OR Denmark OR Finland OR Slovakia OR Norway OR Ireland OR Croatia OR Moldova OR Bosnia OR Herzegovina OR Albania OR Lithuania OR "North Macedonia" OR Slovenia OR Latvia OR Estonia OR Montenegro OR Luxembourg OR Malta OR Iceland OR Andorra OR Monaco OR Liechtenstein OR "San Marino" OR "Holy See")

**Scopus**:

**TITLE-ABS-KEY**((refugee* OR "asylum seeker" OR asylum OR migrant* OR "forced migration" OR migration OR immigra* OR stateless OR "state-less" OR "residence status" OR "foreign-born" OR "noncitizen" OR outsider OR newcomer OR "newly arrived" OR "new arrival" OR "recent entrant" OR "non-national" OR "transient") AND (racism OR racial OR racialization OR racialisation OR discrimination) AND (healthcare OR "dental care" OR patients OR "health care" OR hospitals OR "emergency service" OR health OR "medical staff" OR "health staff") AND (Europe OR European OR Germany OR "United Kingdom" OR France OR Italy OR Spain OR Ukraine OR Poland OR Romania OR Netherlands OR Belgium OR "Czech Republic" OR Czechia OR Greece OR Portugal OR Sweden OR Hungary OR Belarus OR Austria OR Serbia OR Switzerland OR Bulgaria OR Denmark OR Finland OR Slovakia OR Norway OR Ireland OR Croatia OR Moldova OR Bosnia OR Herzegovina OR Albania OR Lithuania OR "North Macedonia" OR Slovenia OR Latvia OR Estonia OR Montenegro OR Luxembourg OR Malta OR Iceland OR Andorra OR Monaco OR Liechtenstein OR "San Marino" OR "Holy See"))

**Web of Science**:

**TS=**((refugee* OR "asylum seeker" OR asylum OR migrant* OR "forced migration" OR migration OR immigra* OR stateless OR "state-less" OR "residence status" OR "foreign-born" OR "noncitizen" OR outsider OR newcomer OR "newly arrived" OR "new arrival" OR "recent entrant" OR "non-national" OR "transient") AND (racism OR racial OR racialization OR racialisation OR discrimination) AND (healthcare OR "dental care" OR patients OR "health care" OR hospitals OR "emergency service" OR health OR "medical staff" OR "health staff") AND (Europe OR European OR Germany OR "United Kingdom" OR France OR Italy OR Spain OR Ukraine OR Poland OR Romania OR Netherlands OR Belgium OR "Czech Republic" OR Czechia OR Greece OR Portugal OR Sweden OR Hungary OR Belarus OR Austria OR Serbia OR Switzerland OR Bulgaria OR Denmark OR Finland OR Slovakia OR Norway OR Ireland OR Croatia OR Moldova OR Bosnia OR Herzegovina OR Albania OR Lithuania OR "North Macedonia" OR Slovenia OR Latvia OR Estonia OR Montenegro OR Luxembourg OR Malta OR Iceland OR Andorra OR Monaco OR Liechtenstein OR "San Marino" OR "Holy See"))

**CINAHL**:

((refugee* OR "asylum seeker" OR asylum OR migrant* OR "forced migration" OR migration OR immigra* OR stateless OR "state-less" OR "residence status" OR "foreign-born" OR "noncitizen" OR outsider OR newcomer OR "newly arrived" OR "new arrival" OR "recent entrant" OR "non-national" OR "transient") AND (racism OR racial OR racialization OR racialisation OR discrimination) AND (healthcare OR "dental care" OR patients OR "health care" OR hospitals OR "emergency service" OR health OR "medical staff" OR "health staff") AND (Europe OR European OR Germany OR "United Kingdom" OR France OR Italy OR Spain OR Ukraine OR Poland OR Romania OR Netherlands OR Belgium OR "Czech Republic" OR Czechia OR Greece OR Portugal OR Sweden OR Hungary OR Belarus OR Austria OR Serbia OR Switzerland OR Bulgaria OR Denmark OR Finland OR Slovakia OR Norway OR Ireland OR Croatia OR Moldova OR Bosnia OR Herzegovina OR Albania OR Lithuania OR "North Macedonia" OR Slovenia OR Latvia OR Estonia OR Montenegro OR Luxembourg OR Malta OR Iceland OR Andorra OR Monaco OR Liechtenstein OR "San Marino" OR "Holy See"))
