# Supplementary material for: Racism against racialized migrants in healthcare in Europe: a scoping review
Source: Int J Equity Health. 2023 Sep 29;22:201. doi: 10.1186/s12939-023-02014-1 (PMC10540333; doi:10.1186/s12939-023-02014-1)
Supplement: Supplementary file 2 — Additional file 2: Supplementary file 2. Overview of the included literature. [file 12939_2023_2014_MOESM2_ESM.docx]

| **Author** | **Year** | **Title** | **Country** | **Aim** | **Method** | **Study Population** | **Main Findings** |
| --- | --- | --- | --- | --- | --- | --- | --- |
| **Qualitative studies** | | | | | | | |
| Akhavan, S | 2012 | Midwives’ views on factors that contribute to health care inequalities among immigrants in Sweden: A qualitative study | Sweden | To explore the views of midwives on the factors that contribute to health care inequality among immigrants | Semi-structured interviews | Healthcare providers: n=10 native Swedish midwives | Midwives noted that migrant women did not receive the same treatment and care as native Swedish women, perhaps due to language barriers, or “ignorance or prejudices” on behalf of the health care services. |
| Akhavan S; Karlsen S | 2013 | Practitioner and client explanations for disparities in health care use between migrant and non-migrant groups in Sweden: a qualitative study | Sweden | To investigate variations in explanations given for disparities in health care use between migrant and non-migrant groups, by clients and care providers in Sweden | Interviews | Healthcare users and healthcare providers: n=5 health service users from Cuba, Russia, Palestine, Bosnia, Iran, and n=5 physicians | Patients believed that healthcare workers often relied on stereotypes when interacting with them, and many had healthcare experiences that left them wondering if the treatment approaches offered to them were affected by their migrant status. Physicians commented that migrants over-emphasized their symptoms to exaggerate their pain, and perceived their lifestyles as detrimental to health and difficult to change. Physicians also expressed concern that foreign patients did not follow the treatment regimes correctly due to cultural differences, saying that changing their habits “requires a bit more force.” One physician said that interpersonal discrimination “may be due to nursing staff that are not open-minded and afraid of something they do not recognize.” |
| Arrey, AE; Bilsen, J; Lacor, P; Deschepper, R | 2017 | Perceptions of stigma and discrimination in health care settings towards Sub-Saharan African migrant women living with HIV/AIDS in Belgium: a qualitative study | Belgium | To explore the causes, forms and consequences of HIV-related stigma and discrimination among migrant sub-Saharan African women living with HIV in Belgium | Semi-structured, in-depth interviews and observations | Healthcare users: n=44 Sub-Saharan African migrant women | Participants said that even within a healthcare setting, HIV was perceived as a “foreign disease”, and they felt blamed for its spread. Many said that people believe that African men and women have many sexual partners. |
| Bawadi H; Al-Hamdan Z; Ahmad MM | 2020 | Needs of Migrant Arab Muslim Childbearing Women in the United Kingdom | United Kingdom | To examine the challenges faced by migrant Arab Muslim women in accessing maternity services and to suggest ways to improve the childbirth experience for them | Semi-structured interviews | Healthcare users: n=8 Arab Muslim women | Some of the participants felt angry about how they had been discriminated against by their midwives. One woman described being spoken to rudely by a midwife who behaved differently with the British patient next door. Other women reported that the midwives perceived them as demanding and complaining. In postnatal wards, the women said that they did not receive help from the staff and felt mistreated. |
| Bird, R; Oezer-Erdogdu, I; Aslan, M; Tezcan-Guentekin, H | 2022 | Healthcare Provider Perspectives on Digital and Interprofessional Medication Management in Chronically Ill Older Adults of Turkish Descent in Germany: A Qualitative Structuring Content Analysis | Germany | To examine health care provider perspectives on interprofessional cooperation and digital medication management tools as approaches for increasing medication safety for chronically ill older adults of Turkish descent in Germany | Semi-structured interviews | Healthcare providers: n=11 providers of chronically ill older adults of Turkish descent | Several participants reported witnessing incidents of racism toward older adults of Turkish descent in the healthcare system. One of the respondents reported their own prejudiced views, stating: “It’s exhausting... Let’s say a female patient of Turkish descent comes in and she’s quite hysterical – in my perception. Then it would take me ten minutes to first of all talk her down from that state of hysteria. That’s really a lot of work for me. And when I feel stressed, it makes me aggressive.” The same respondent stated that they have no interest in utilizing training opportunities for diversity sensitivity, citing an “aggressive psychological barrier” as a justification. |
| Bollini P; Stotzer U; Wanner P | 2007 | Pregnancy outcomes and migration in Switzerland: results from a focus group study | Switzerland | To explore the issues of pregnancy and delivery in migrant women in their interaction with the Swiss healthcare system | Focus groups | Healthcare users: n=40 women, including 14 Turkish, 17 Portuguese, and 9 Swiss | Turkish women described experiencing a sense of discrimination in their encounter with the healthcare system, as they felt the needs of foreign women were seen less sensitively as compared to their Swiss counterparts. |
| Chaouni, SB; Smetcoren, A-S; De Donder, L | 2020 | Caring for migrant older Moroccans with dementia in Belgium as a complex and dynamic transnational network of informal and professional care: A qualitative study | Belgium | To explore how dementia care is provided to these Moroccan older people with dementia, and what challenges caregivers face in providing care | Focus groups | Healthcare users and healthcare providers: n=6 informal and professional caregivers, n=12 informal caregivers of Moroccan decent and n=13 professional caregivers | Some informal caregivers of Moroccan patients described experiencing discrimination and racism during their healthcare encounters. One informal caregiver formulated: “You should have seen the way how he (a doctor) talked to me and to my father. Without any respect, like we were animals…it hurts me that my father has to endure such racism at this stage of his life.” |
| Erwin, J.; Peters, B. | 1999 | Treatment issues for HIV+ Africans in London | United Kingdom (London) | To explore the treatment issues concerning black Africans which may affect their uptake of therapies | Focus groups | Healthcare users: n=44 migrants from Uganda, Zambia, Ethiopia, Nigeria, Kenya, Zimbabwe and Tanzania | Some participants felt that Africans were pressured to take medication and not given the opportunity to participate in treatment decisions, while others described the failure of doctors to tell them what to do about treatment. They also expressed their beliefs that black African patients are not well looked after and that doctors actually hastened the death of African patients. For instance, five of six focus groups mentioned the refusal of medical staff to feed the patients when they got ill, and two groups perceived the intentional killing of their relatives through drugs. |
| Kang, C; Tomkow, L; Farrington, R | 2019 | Access to primary health care for asylum seekers and refugees: a qualitative study of service user experiences in the UK | United Kingdom | To examine asylum-seeker and refugee experiences accessing primary health care in the UK in 2018 | Semi-structured interviews | Healthcare users: n=18 asylum-seekers and refugees from Pakistan, Sudan, Syria, Iran, Libya, Eritrea, Ivory Coast, and Guinea | Several participants reported experiencing discrimination from staff at the surgery owing to their race, religion, or immigration status. One staff member was described as being “really, really racist” through her body language, and another receptionist was said to prioritize patients who were not wearing a hijab. |
| Kikhia S; Gharib G; Sauter A; Vincens NCL; Loss J | 2021 | Exploring how Syrian women manage their health after migration to Germany: results of a qualitative study | Germany | To describe how Syrian immigrant women in Germany feel about their capabilities to manage their health and that of their families, and to explore the social and environmental circumstances of Syrian migrated women, that act as barriers and/or facilitators to effectively manage health | In-depth interviews | Healthcare users: n=9 Syrian migrant women | Most of the interviewed women felt that their health complaints were not being taken seriously or that they were not properly examined or treated by the healthcare provider, and often considered this a sign of neglect, prejudice, or discrimination. One woman with back pain was told by a doctor that it was psychosomatic. He said that “You Syrians have gone through a lot of stress,” and did not order an X-ray. The woman said that she doesn’t believe Germans would be fine with being treated like that. |
| Kour, P; Lien, L; Kumar, B; Biong, S; Pettersen, H | 2020 | Treatment Experiences with Norwegian Health Care among Immigrant Men Living with Co-Occurring Substance Use- and Mental Health Disorders | Norway | To explore the treatment experiences of immigrant men living with co-occurring SUD and MHD | Interviews | Healthcare users: n=10 men with co-occurring SUD and MHD; five participants were from the Middle East, while others were from South Asia and East and West Africa | A majority of participants experienced a lack of interest from health professionals, as well as not being seen as a person, while they were in treatment. In addition, participants recounted experiences of discrimination that they faced, including incidents of being looked down upon by the health providers. One participant described feeling very hurt, labeled, and judged when they started sharing racist incidents in their life with a psychiatrist, who only responded with, “Are you violent?” |
| Lafaut, D; Coene, G | 2020 | "I was trying to speak to their human side" coping responses of Belgium's undocumented migrants to barriers in health-care access | Belgium | To explore the coping responses of undocumented migrants when they experience limited healthcare access in face-to-face encounters with healthcare providers | Multisite ethnographic observations and semi-structured interviews | Healthcare users; n=25 undocumented migrants from Sub-Saharan Africa, North Africa, the Middle East, South Asia and South America | An undocumented migrant who asked to see the ophthalmologist noticed that the native Belgian patient in front of him was getting a much quicker appointment than him. One respondent described having to deliberately avoid healthcare workers that look or are known to be obstructive, such as a social assistant who had mistreated him. One respondent said that staff from an ambulance-team refused to take an undocumented friend to the hospital, after finding out he had no legal residence status. |
| Lien IL | 2021 | Health workers and Sub Saharan African women's understanding of equal access to healthcare in Norway | Norway | To find out if there is equal access to healthcare as understood by both the provider and receiver side of healthcare | Interviews | Healthcare users and healthcare providers: n=55 migrant women from Somali, Gambia and Eritrea women who all had experienced female genital mutilation/cutting, and health workers | Some health workers said other health workers talk behind patients’ backs and stigmatize women with migrant backgrounds such as women from African countries. Though most participants did not think that there was discrimination within the healthcare system, 2 of the 55 interviewees had heard of discrimination against migrants, though they had not experienced it themselves. One woman said that she had experienced it herself. |
| Likupe, G; Baxter, C; Jogi, M | 2018 | Exploring health care workers' perceptions and experiences of communication with ethnic minority elders | United Kingdom | To explore health care workers’ perceptions and experiences of communication with ethnic minority elders | Semi-structured interviews | Healthcare providers: n=10 healthcare workers | Some participants reported stereotyping of patients by health care workers as ethnic minorities from a particular culture and therefore needing a specific form of care, and felt that this was a barrier to communication. |
| Liu, CH; Meeuwesen, L; van Wesel, F; Ingleby, D | 2015 | Why do ethnic Chinese in the Netherlands underutilize mental health care services? Evidence from a qualitative study | Netherlands | To investigate the experiences of Chinese with mental health problems, to inform measures to make services more responsive to the needs of this group | Interviews | Healthcare users: n=25 Chinese patients | Participants described experiences of discrimination as having a negative effect on the quality of treatment. One Chinese woman’s treatment (psychotherapy) remained a struggle until she got help from a psychiatrist originating from the same country. She thought the care was terrible, and said the health professionals made a clear distinction between people from the East and full-blooded Dutch. |
| Lyons SM; O'Keeffe FM; Clarke AT; Staines A | 2008 | Cultural diversity in the Dublin maternity services: the experiences of maternity service providers when caring for ethnic minority women | Ireland | To explore the experiences, understanding and perspectives of maternity service providers when working with ethnic minority women in the Dublin maternity services during 2002 and 2003 | Focus groups and semi-structured interviews | Healthcare providers: n=15 obstetricians, midwives, and auxiliary nurses | Participants observed that some ethnic minority women were thought to be louder, “more dramatic,” “noisy ones,” and consequently more difficult to deal with in labor as they were perceived to require more than their “fair share” of attention. There was also some generalization by participants that many ethnic minority women preferred not to have pain relief during labor, which was sometimes seen negatively as it added to their workload. The “them versus us” negative comparison was a theme that came through the language used to refer to Irish women “versus” ethnic minority women. Service providers often expressed negative feelings when talking about ethnic minority women, as “stress”, “worry”, and “difficult” recurred throughout the texts. Other words that also appeared were frustration and tired/exhausted. Some participants spoke about ethnic minority women’s reasons for coming to Ireland, querying their legitimacy or whether they were “genuine” or not. Some had seen interactions that they felt to be unacceptable and racist from both other staff and patients in the hospital. Some service providers considered that they were not racist but felt they had genuine reasons for their issues in relation to ethnic minority women: “Because they’re in our face all the time and they’re rude to us. I’m not racist, it’s very frustrating ... if they were genuine [asylum seekers] ....” Even though 58% of ethnic minority women were not African, some participants often referred to all ethnic minority women as African when generalizing. One also commented that Irish women “come to a hospital in their own country and there’s maybe more blacks than whites” |
| May, P | 2021 | The Letter and Spirit of the Law: Barriers to Healthcare Access for Asylum Seekers in France | France | To focus on the barriers to healthcare access experienced by asylum seekers in France | Interviews | Healthcare users and healthcare providers: n=40 Sub-Saharan African asylum seekers who used health services, n=12 asylum seekers who did not use health services, n=7 medical, NGO and associative staff | Several participants suggested that, during medical appointments, doctors tend to devote less time to refugees than to other patients. Eight participants were rejected by medical specialists despite the fact that they were eligible for free treatment. Three participants claimed to have received remarks that were clearly defamatory, targeting their ethnic or national origin. One patient was told that he “was being taking care of as a favor, and that [he] had to do [his] part of the job and contribute to social security once [he] had acquired the residence permit. One man was told by a nurse that refugees “brought diseases like polio that had disappeared in Europe a long time ago.” |
| Mbanya, VN; Terragni, L; Gele, AA; Diaz, E; Kumar, BN | 2019 | Access to Norwegian healthcare system - challenges for sub-Saharan African immigrants | Norway | To explore the experiences of Sub-Saharan African immigrants in accessing the Norwegian healthcare services | Two focus group discussions and interviews | Healthcare users: n=47  migrants from Sub-Saharan Africa | Most migrants preferred doctors with a migrant background, particularly from Africa, due to the respect, attention, and treatment they perceived to get. Many participants felt discouraged because they perceived the care providers did not seem interested in them. They felt ignored and treated as second-class citizens, and believed the care providers paid less attention to them than they did to other patients of a different race. One patient said, “They pretend not to understand you [someone] and they ignore your presence and concentrate on different patients that are white.” Some participants also felt the care providers were scrutinizing them and asking questions concerning their private life, “like what brought me to this country and why don’t I go back to Uganda and find a better job.” One patient said that doctors immediately assume they are there for sick leave. Most of the participants noted that HIV test was often among the list of laboratory test checks, and it was obvious they were being suspected of having infectious diseases, which were affirmed by the facial expression and actions of the healthcare providers. Several participants perceived that providers did not want to have direct skin contact with them, through facial expressions or actions such as double-gloving. Most believed that this discrimination was because they were black and from Africa, and participants perceived this to be disrespectful, unfriendly and an idea that has been preconceived of Africans. They felt neglected and isolated and supposed the healthcare providers preferred talking to patients of different ethnic background and race. One patient noted that their nurse ignored them during dialysis and instead was paying attention to a nearby Norwegian patient. One patient said that, “The doctors treat the African differently. We are not always greeted in a friendly manner as compared […] the people are always biased towards Africans. They treat us different and they talk to other white people with respect, but with us, they are very rigid. They do not smile and only send us to do the test or prescribed medications. No physical examination.” |
| Moleiro, C.; Freire, J.; Tomsic, M. | 2013 | Immigrant perspectives on multicultural competencies of clinicians: A qualitative study with immigrants in Portugal | Portugal | To explore the experiences of individuals of ethnic minority groups regarding their access to the Portuguese healthcare system and to identify the multicultural competencies of the clinicians (as perceived by the clients) which would be required to improve culturally sensitive treatments | Eight focus groups and semi-structured interviews | Healthcare users: n=40 adults from African countries, Brazil, and other European countries | The frequency of discriminatory experiences among patients was in the “general” category, the highest frequency of three categories. All participants had undergone prior discriminatory experiences within the healthcare system. |
| Nelson M; Patton A; Robb K; Weller D; Sheikh A; Ragupathy K; Morrison D; Campbell C | 2021 | Experiences of cervical screening participation and non-participation in women from minority ethnic populations in Scotland | Scotland | To explore experiences of cervical screening participation and nonparticipation of women from minority ethnic populations in Scotland and gain insights to support the development of interventions that could potentially support screening participation and thereby reduce inequalities | Semi-structured interviews and qualitative comparison study | Healthcare users: n=50 participants from South Asia, East Europe, China, Africa, Carribean, and Scotland | Participants in the Chinese, South Asian and Black African/ Caribbean groups talked of difficulties related to ignorance and racism from both clinical and non-clinical staff. One South Asian patient said that the comments made were quite demeaning. One Chinese participant said that “There was a racial thing going on there. Because it wasn’t just me, it was any colored person that went in.” Participants in the Black African/Caribbean group talked of a lack of practitioners’ understanding of normal and abnormal physiology in Black and minority populations. |
| Perez-Urdiales, I; Goicolea, I; San Sebastian, M; Irazusta, A; Linander, I | 2019 | Sub-Saharan African immigrant women's experiences of (lack of) access to appropriate healthcare in the public health system in the Basque Country, Spain | Spain | To analyze Sub-Saharan African immigrant women's perceptions and experiences of access to appropriate healthcare in the public health system in the Basque Country, Spain | Interviews | Healthcare users: n=14 women from eight Sub-Saharan African countries (Cameroon, Guinea, Bissau, Senegal, Angola, Democratic Republic of Congo, Gambia, Nigeria) | Participants said that professionals treated them poorly and had poor efforts to communicate with them, due to their identity as a migrant. Some participants expressed having received differentiated treatment or mistreatment, as perceived in the way they behaved compared with migrant women compared to natives. Two participants who worked at social organizations made a connection between the poorer attention given to black people in the health system with historical colonial domination of high-income countries, stating that the image of Africa presented in the mass media as a homogeneous land of corruption, poverty and famine reinforces a negative collective social imaginary on Africans and black people shared by the general population and by professionals working at the health system. One said that “There is an imaginary that blacks are like animals, uneducated, rude, primary, ignorant… All those labels that the history has accumulated on us influence the way we are attended” |
| Robertson, EK | 2015 | "To be taken seriously" : women's reflections on how migration and resettlement experiences influence their healthcare needs during childbearing in Sweden | Sweden | To use an intersectional approach to analyze women’s reflections on how their migration and resettlement experiences to Sweden influenced their health and healthcare needs during childbearing | Focus-group discussions, pair interviews and individual interviews | Healthcare users: n=25 women from 17 different countries (Bosnia, Chile, China, El Salvador, Ethiopia, Eritrea, Iran, Iraq, Kosovo, Lebanon, Morocco, Slovenia, Spain, Syria, Turkey, Uzbekistan, and former Yugoslavia) | Most of the women expressed that when they felt uncared for or ignored during pregnancy, miscarriage, or childbirth, they felt disappointed and discriminated against. They stated that being treated like they were children or legally incompetent made them feel stupid and doubt their capabilities. All the interviewed women shared experiences of being met with distrust and prejudices in caring encounters. Some women felt ignored, lacked a caring response to their worries, and were treated as if they were exaggerating. The women stated that not receiving the same kind of attention as Swedish mothers constituted discrimination. |
| Schein, YL; Winje, BA; Myhre, SL; Nordstoga, I; Straiton, ML | 2019 | A qualitative study of health experiences of Ethiopian asylum seekers in Norway | Norway | To describe the health-related experiences of Ethiopians who have sought asylum in Norway and shed light on potential barriers to care | Interviews | Healthcare users: n=10 Ethiopian refugees and asylum seekers | Issues of race complicated interactions with doctors, as participants indicated a difference in their care based on the fact that they were not Norwegian. Participants described Norwegian-born friends receiving more details from their doctor about treatments, and having seen doctors who have been dismissive towards them act much friendlier with Norwegian patients, theoretically “because some of them don’t like non-Norwegians or Africans”. Multiple participants described better healthcare interactions with doctors who were perceived as also not Norwegian. |
| Schmidt, NC; Fargnoli, V; Epiney, M; Irion, O | 2018 | Barriers to reproductive health care for migrant women in Geneva: a qualitative study | Switzerland | To explore barriers to reproductive health services in Geneva described by migrant women from a qualitative perspective | Focus groups | Healthcare users: n=78 women from Eritrea, Albania, the Philippines, the Middle East and Latin-America | Nearly one-third of the participants expressed that they felt that they did not receive the same attendance as Swiss women, mainly concerning reception at the registration desk prior to the clinical appointment who asked for papers, i.e. proof of health insurance, which made them feel degraded. A few women expressed that waiting time as well as perceived impolite treatment by health professionals were due to their origins or language barriers. |
| Scott, P | 2014 | Black African asylum seekers' experiences of health care access in an eastern German state | Germany | To examine how access to health care for (rejected) asylum seekers in an eastern German state is structured and experienced and to consider the implications for their human rights | Interviews | Healthcare users: n=12 rejected black African asylum seekers | Participants cited discrimination such as withholding of care to which they were entitled and standards not being followed. One patient said that she could see that she did not receive the attention that other women received in the hospital, while another said that “Some of the doctors, especially when you are black and a foreigner, they don’t care.” They reported that during the consultation they were often spoken to in a “rude” or condescending manner and experienced rejecting behaviors. Several participants complained that doctors’ had asked “prying questions” about their asylum claim, and one participant added that such questions were racist: they appeared rooted in prejudiced assumptions about asylum seekers and served to reproduce their devalued social identity. Some expressed consciousness of being at the bottom of an ethno-racial hierarchy of asylum seekers, as their lighter skinned and eastern Europeans counterparts were described as benefiting from certain privileges and exemptions whereas “everything that is black is bad and criminal”. |
| Worth A; Irshad T; Bhopal R; Brown D; Lawton J; Grant E; Murray S; Kendall M; Adam J; Gardee R; Sheikh A | 2009 | Vulnerability and access to care for South Asian Sikh and Muslim patients with life limiting illness in Scotland: prospective longitudinal qualitative study | Scotland | To examine the care experiences of South Asian Sikh and Muslim patients in Scotland with life limiting illness and their families and to understand the reasons for any difficulties with access to services and how these might be overcome | Interviews | Healthcare users and healthcare providers: n=25 South Asian Sikh and Muslim patients, n=18 family carers, and n=20 key health professionals | Patients, family carers, and professionals expressed that barriers arose from reasons including perceptions and beliefs of ethnic minority communities, as well as the attitudes of service providers. Patients and families sometimes perceived prejudice from service providers in terms of rudeness, lack of sensitivity, or lack of interest. One Muslim patient said that a staff member kept asking when he would be taken home, adding that “I’m sure if he was white, nobody would have said that, take him home.” A Sikh patient with multiple long term conditions, who was homeless and an asylum seeker, complained of rude, hostile attitudes by staff, neglect of his needs, continually receiving inappropriate food, and feeling humiliated by staff hostility if he complained about the food or asked questions about his treatment, as one nurse said “I will paint a horrible picture of you and report you to the immigration and they will deport you.” Another patient described how Asian staff advised him to keep smiling all the time, saying that “you should be grateful because you are receiving free of charge treatment which would not be possible in [own country].” One professional interviewed confirmed that a patient’s care had been poor, his dietary needs unmet, and his treatment discriminatory, saying that some clinicians and managers suggested “that they send him back to [own country] as soon as they possibly could, apparently without any notion of the consequences.” |
| **Mixed-Methods studies** | | | | | | | |
| Sauvegrain, P; Azria, E; Chiesa-Dubruille, C; Deneux-Tharaux, C | 2017 | Exploring the hypothesis of differential care for African immigrant and native women in France with hypertensive disorders during pregnancy: a qualitative study | France | To analyze whether prenatal care trajectories among women with hypertensive disorders during pregnancy in France differ between immigrants from sub-Saharan Africa and native French women | Interviews and collected data from medical files | Healthcare users: n=33 Sub-Saharan African women | Results suggest non-medically justified differential prenatal care between African and native women that may have helped delay the diagnosis of hypertension or preeclampsia. Women reported that the blood pressure measurement procedures used by hospital staff varied between the two groups, and their medical records supported this finding. Repeated urinary dipstick testing of proteinuria before laboratory testing was more frequent for African women, as was the failure to further test proteinuria levels requiring additional action. The differences found involved, in particular, screening tests that were more often repeated before moving on to confirmation by diagnostic tests, more frequent failure to take action for values on the borderline of abnormal, as well as the prevention messages provided to women to recognise the occurrence of the disease being non-existent or too simplified. This delayed the diagnosis of HBP or pre-eclampsia in African women, especially those with hypertension for the first time. |
| **Quantitative studies** | | | | | | | |
| Farrukh A; Mayberry J | 2016 | Patients with ulcerative colitis from diverse populations: The Leicester experience | England | To investigate whether South Asians experienced the same quality of care as English patients with ulcerative colitis | Retrospective review of case notes | Healthcare users: n=42 English and n=28 South Asian patients newly diagnosed with ulcerative colitis | Patients with ulcerative colitis who are of South Asian origin receive poorer quality clinical care than their European counterparts. South Asian patients were significantly less likely to be reviewed by a consultant and more likely to be discharged. South Asian patients were admitted to hospital more often but had significantly fewer tests than European patients. |
| Farrukh A; Mayberry J | 2020 | Apparent Disparities in Hospital Admission and Biologic Use in the Management of Inflammatory Bowel Disease between 2014-2018 in Some Black and Ethnic Minority (BEM) Populations in England | England | To explore discrimination in delivery of care to patients with inflammatory bowel disease for Afro-Caribbean and Eastern European migrant workers | Retrospective analysis of data on use of biologics or hospital admissions over a five-year period | Healthcare users: n=3,382,394 Afro-Caribbean and Eastern European migrant workers | Ten Trusts provided data on hospital admissions. In Bristol, Nottingham, Derby and Burton, Princess Alexandra Hospital Trust in Harlow, Essex and Kings College Hospital NHS Foundation Trust in South London Afro-Caribbean patients were treated significantly less often than White British patients. In North West Anglia both South Asian and Eastern European patients were significantly less likely to be admitted to hospital than members of the White British community. |
| Fassaert, T; Peen, J; van Straten, A; de Wit, M; Schrier, A; Heijnen, H; Cuijpers, P; Verhoeff, A; Beekman, A; Dekker, J | 2010 | Ethnic Differences and Similarities in Outpatient Treatment for Depression in the Netherlands | Netherlands | To address ethnic differences in characteristics of outpatient treatment for depression in the Netherlands | Retrospective analysis of longitudinal data from a nationwide psychiatric case register | Healthcare users: n=17,270 Dutch and non-Dutch patients | Timeliness and treatment intensity were somewhat less favorable for Moroccan, Turkish, and other non-Western clients compared with ethnic Dutch. |
| Knutzen, M.; Sandvik, L.; Hauff, E.; Opjordsmoen, S.; Friis, S. | 2007 | Association between patients' gender, age and immigrant background and use of restraint - A 2-year retrospective study at a department of emergency psychiatry | Norway | To determine rates and types of patient restraint, and their relationship to age, gender and immigrant background | Retrospective analysis of n=960 patients’ data in a department of acute psychiatry over a 2-year period | Healthcare users: n=960 migrant and native patients | Patients with migrant background were both more often and more heavily restrained. Most commonly used were mechanical restraint alone for native-born patients and a combination of mechanical and pharmacological restraints for patients with migrant background. |
| Nielsen, TR; Andersen, BB; Kastrup, M; Thien, KTP; Waldemar, G | 2011 | Quality of Dementia Diagnostic Evaluation for Ethnic Minority Patients: A Nationwide Study | Denmark | To evaluate the quality of diagnostic evaluation of dementia for patients from ethnic minorities in Denmark | Retrospective analysis of data from the Danish national hospital registers | Healthcare users: n=57 immigrants from Turkey, Pakistan, or ex-Yugoslavia who were diagnosed with dementia in the period 2005–2007 | Significant differences in the quality of the diagnostic evaluation were found between patients from ethnic minorities and the general population. |
| Cilenti, K; Rask, S; Elovainio, M; Lilja, E; Kuusio, H; Koskinen, S; Koponen, P; Castaneda, AE | 2021 | Use of Health Services and Unmet Need among Adults of Russian, Somali, and Kurdish Origin in Finland | Finland | To analyze differences in the use of health services between people of migrant origin and the general population | Cross-sectional study of the Health 2011 survey data | Healthcare users: n=692 Russian patients, n=489 Somali patients, n=614 Kurdish patients, and n=1406 patients representing the general population in Finland | Compared to the general population, all migrant origin groups reported much higher levels of unmet medical need and were less satisfied with the treatment they had received. All groups of migrant origin, except men of Somali origin, agreed less often than the general population that the access was quick, that they received enough information, were being listened to or that the treatment helped them. |
| Gil-Salmerón A; Katsas K; Riza E; Karnaki P; Linos A | 2021 | Access to Healthcare for Migrant Patients in Europe: Healthcare Discrimination and Translation Services. | 10 European Union countries (Austria, Bulgaria, Cyprus, France, Germany, Greece, Italy, Malta, Spain, and Sweden) | To assess healthcare discrimination as perceived by migrants themselves and the availability of translation services in the healthcare systems of Europe | Cross sectional survey analysis measuring discrimination using the DMS scale and descriptive analysis | Healthcare users: n=1294 participants, primarily from Syria, Afghanistan, Iraq, Nigeria, and Iran | A high DMS score (indicating more perceived discrimination) was observed in Greece, Italy, Cyprus, and Austria, with migrants from Afghanistan tending to score higher. Women from Iran scored significantly higher in the DMS scale compared with other countries of origin. Older migrants reported higher feelings of health discrimination. |
| Pacheco, LL; Jonzon, R; Hurtig, AK | 2016 | Health Assessment and the Right to Health in Sweden: Asylum Seekers' Perspectives | Sweden | To assess how the information, procedures and services related to the health assessment are accessible and acceptable to fulfill the right to health of asylum seekers, from their own perspective | Cross-sectional questionnaire | Healthcare users: n=386 patients from Somalia, Syria, Eritrea, Afghanistan, Iraq, and other countries | The health assessment is the first encounter with the Swedish health care system for most asylum applicants, and has the purpose to identify health problems demanding immediate attention in the best interest of the individual and to identify and take appropriate measures to prevent the spread of contagious diseases as a public health measure. 37 (10.9%) survey respondents expressed distrust in the person who carried out the health assessment and indicated feeling offended or insulted. They considered being treated inappropriately due to their language difficulties, having another ethnic, religious or cultural background, or because of gender and age differences with the health care provider. |
| Rivenbark, JG; Ichou, M | 2020 | Discrimination in healthcare as a barrier to care: experiences of socially disadvantaged populations in France from a nationally representative survey. | France | To examine social disparities in discrimination within healthcare, foregone healthcare, and how they are related | Cross-sectional analysis of survey conducted via in-person interviews and coded dichotomously | Healthcare users: n=21,761 patients, with an oversample of immigrants | Rates of both reporting discrimination within healthcare and reporting foregone care in the past 12 months were generally highest among women, migrants from Africa or Overseas France, and Muslims. For all of these groups, experiences of discrimination potentially explained significant proportions of their disparity in foregone care. |
| Duveau C; Demoulin S; Dauvrin M; Lepièce B; Lorant V | 2022 | Implicit and explicit ethnic biases in multicultural primary care: the case of trainee general practitioners | Belgium | To investigate the extent of implicit ethnic biases and willingness to adapt care to migrant patients among trainee GPs, and the factors involved therein, in order to measure explicit bias and explore a dimension of cultural competence | Prospective study using the Implicit Association Test (IAT) to measure implicit biases against ethnic groups and the Hudelson scale, used to assess an explicit attitude of willingness to adapt care to diversity | Healthcare providers: n=207 practitioners | The test found a moderate association of positive words with French language first names over North African first names. A general practitioner being of Arab ethnicity or of other or mixed ethnic origin reduced the strength of implicit negative ethnic associations with North African first names, as did having left-wing political opinions. |
